# Supplementary material for: CXCL12 Gene Polymorphisms and Serum Levels: Associations with Multiple Sclerosis Prevalence and Clinical Parameters in Lithuania
Source: Int J Mol Sci. 2024 Sep 3;25(17):9554. doi: 10.3390/ijms25179554 (PMC11395108; doi:10.3390/ijms25179554)
Supplement: Supplementary file 1 [file ijms-25-09554-s001.zip › ijms-3139799-supplementary.pdf]

Supplementary Materials

Table S1. Age-Stratified Distribution of *CXCL12* (rs1029153, rs1801157 and rs2297630) Genotypes and Alleles in Multiple Sclerosis and Control Groups

***CXCL12* SNP**	**< 40 years old**				**≥ 40 years old**			
	**Genotype / allele**	**Control Group n (%)**	**MS group n (%)**	**p value**	**Genotype / allele**	**Control Group n (%)**	**MS group n (%)**	**p value**
rs1029153	AA	65 (51.6)	73 (57.9)	0.335	AA	59 (47.6)	59 (47.6)	1.000
	AG	51 (40.5)	40 (31.7)		AG	51 (41.1)	51 (41.1)	
	GG	10 (7.9)	13 (10.3)		GG	14 (11.3)	14 (11.3)	
	A allele	181 (71.8)	186 (73.8)	0.468	A allele	169 (68.1)	169 (68.1)	1.000
	G allele	71 (28.2)	66 (26.2)		G allele	79 (31.9)	79 (31.9)	
rs1801157	CC	67 (53.2)	85 (67.5)	0.068	CC	85 (68.5)	81 (65.3)	0.362
	CT	53 (42.1)	37 (29.4)		CT	35 (28.2)	34 (27.4)	
	TT	6 (4.8)	4 (3.2)		TT	4 (3.2)	9 (7.3)	
	C allele	181 (71.8)	207 (82.1)	0.022	C allele	205 (82.7)	196 (79)	0.468
	T allele	71 (28.2)	45 (17.9)		T allele	43 (17.3)	52 (21)	
rs2297630	GG	66 (52.4)	74 (58.7)	0.570	GG	63 (50.8)	65 (52.4)	0.838
	AG	52 (41.3)	44 (34.9)		AG	45 (36.3)	46 (37.1)	
	AA	8 (6.3)	8 (6.3)		AA	16 (12.9)	13 (10.5)	
	G allele	181 (71.8)	192 (76.2)	0.353	G allele	171 (69)	176 (71)	0.695
	A allele	71 (28.2)	60 (23.8)		A allele	77 (31)	72 (29)	

SNP – single nucleotide polymorphism, MS – multiple sclerosis. Bonferroni corrected significance level $p < 0.0167$.

Table S2. Age-Stratified Binary Logistic Regression Analysis of *CXCL12* (rs1029153, rs1801157 and rs2297630) Genetic Models

Model	Genotype / allele	Odds Ratio (95 % CI)	p value	AIC
**< 40 years old**				
**Model**	**Genotype / allele**	**Odds Ratio (95 % CI)**	**p value**	**AIC**
***CXCL12* (rs1029153):**				
Codominant	AG vs. AA	0.7 (0.41 - 1.19)	0.186	347.157
	GG vs. AA	1.16 (0.48 - 2.82)	0.747	
Dominant	AG+GG vs. AA	0.77 (0.47 - 1.27)	0.312	348.320
Recessive	GG vs. AA+AG	1.34 (0.56 - 3.17)	0.513	348.914
Overdominant	AG vs. GG+AA	0.68 (0.41 - 1.15)	0.150	347.261
Additive	G	0.91 (0.63 - 1.33)	0.631	349.116
***CXCL12* (rs1801157):**				
Codominant	CT vs. CC	0.55 (0.33 - 0.93)	0.027	343.947
	TT vs. CC	0.53 (0.14 - 1.94)	0.334	
Dominant	CT+TT vs. CC	0.55 (0.33 - 0.91)	0.021	343.952
Recessive	TT vs. CC+CT	0.66 (0.18 - 2.38)	0.521	348.927
Overdominant	CT vs. TT+CC	0.57 (0.34 - 0.97)	0.036	344.904
Additive	T	0.61 (0.39 - 0.95)	0.029	344.423
***CXCL12* (rs2297630):**				
Codominant	AG vs. GG	0.76 (0.45 - 1.27)	0.290	348.221
	AA vs. GG	0.89 (0.32 - 2.51)	0.828	
Dominant	AG+AA vs. GG	0.77 (0.47 - 1.27)	0.311	348.317
Recessive	AA vs. GG+AG	1 (0.36 - 2.75)	1.000	349.346
Overdominant	AG vs. AA+GG	0.76 (0.46 - 1.27)	0.300	348.268
Additive	A	0.85 (0.56 - 1.27)	0.412	348.672
**≥ 40 years old**				
**Model**	**Genotype / allele**	**Odds Ratio (95 % CI)**	**p value**	**AIC**
***CXCL12* (rs1029153):**				
Codominant	AG vs. AA	1 (0.59 - 1.7)	1.000	343.801
	GG vs. AA	1 (0.44 - 2.28)	1.000	
Dominant	AG+GG vs. AA	1 (0.61 - 1.65)	1.000	343.801
Recessive	GG vs. AA+AG	1 (0.46 - 2.2)	1.000	343.801
Overdominant	AG vs. GG+AA	1 (0.6 - 1.66)	1.000	343.801
Additive	G	1 (0.69 - 1.45)	1.000	343.801
***CXCL12* (rs1801157):**				
Codominant	CT vs. CC	1.02 (0.58 - 1.79)	0.947	341.717
	TT vs. CC	2.36 (0.7 - 7.97)	0.166	
Dominant	CT+TT vs. CC	1.16 (0.68 - 1.97)	0.589	343.509
Recessive	TT vs. CC+CT	2.35 (0.7 - 7.84)	0.165	341.721
Overdominant	CT vs. TT+CC	0.96 (0.55 - 1.67)	0.887	343.781
Additive	T	1.24 (0.81 - 1.9)	0.329	342.841
***CXCL12* (rs2297630):**				
Codominant	AG vs. GG	0.99 (0.58 - 1.7)	0.973	343.448
	AA vs. GG	0.79 (0.35 - 1.77)	0.563	
Dominant	AG+AA vs. GG	0.94 (0.57 - 1.54)	0.799	343.736
Recessive	AA vs. GG+AG	0.79 (0.36 - 1.72)	0.553	343.449
Overdominant	AG vs. AA+GG	1.04 (0.62 - 1.74)	0.895	343.784
Additive	A	0.92 (0.64 - 1.32)	0.645	343.588

CI – confidence interval, AIC – Akaike information criteria. Corrected significance level p < 0.0167.

Table S3. Gender-Stratified Genotype and Allele Frequencies of *CXCL12* (rs1029153, rs1801157 and rs2297630) in Control and Multiple Sclerosis Groups

***CXCL12* SNP**	**Women**				**Men**			
	**Genotype / allele**	**Control Group n (%)**	**MS group n (%)**	**p value**	**Genotype / allele**	**Control Group n (%)**	**MS group n (%)**	**p value**
rs1029153	AA	73 (44.2)	84 (50.9)	0.075	AA	51 (60)	48 (56.5)	0.235
	AG	78 (47.3)	59 (35.8)		AG	24 (28.2)	32 (37.6)	
	GG	14 (8.5)	22 (13.3)		GG	10 (11.8)	5 (5.9)	
	A allele	224 (67.9)	227 (68.8)	0.555	A allele	126 (74.1)	128 (75.3)	0.922
	G allele	106 (32.1)	103 (31.2)		G allele	44 (25.9)	42 (24.7)	
rs1801157	CC	109 (66.1)	108 (65.5)	0.861	CC	43 (50.6)	58 (68.2)	0.037
	CT	50 (30.3)	49 (29.7)		CT	38 (44.7)	22 (25.9)	
	TT	6 (3.6)	8 (4.8)		TT	4 (4.7)	5 (5.9)	
	C allele	268 (81.2)	265 (80.3)	0.851	C allele	124 (72.9)	138 (81.2)	0.034
	T allele	62 (18.8)	65 (19.7)		T allele	46 (27.1)	32 (18.8)	
rs2297630	GG	79 (47.9)	90 (54.5)	0.285	GG	50 (58.8)	49 (57.6)	0.179
	AG	72 (43.6)	58 (35.2)		AG	25 (29.4)	32 (37.6)	
	AA	14 (8.5)	17 (10.3)		AA	10 (11.8)	4 (4.7)	
	G allele	230 (69.7)	238 (72.1)	0.367	G allele	125 (73.5)	130 (76.5)	0.81
	A allele	100 (30.3)	92 (27.9)		A allele	45 (26.5)	40 (23.5)	

SNP – single nucleotide polymorphism, MS – multiple sclerosis. Bonferroni corrected significance level $p < 0.0167$.

Table S4. Characteristics of CXCL12 Protein Evaluation Groups

Characteristic	Group		p value
	Control	MS	
Men (%)	17 (44.7)	18 (45.0)	0.981
Women (%)	21 (55.3)	22 (55.0)	
Men median age (IQR)	32 (12)	32.5 (14)	0.883
Women median age (IQR)	36 (25)	33 (27)	0.733

IQR – interquartile range, MS – multiple sclerosis.

Table S5. CXCL12 Serum Protein Analysis by Gender and Age Groups

	Group		Comparison across groups (p value)
	Control	MS	
Men CXCL12 concentration (pg/ml) median (IQR)	37.5 (5.2)	36.2 (13.6)	0.636
Women CXCL12 concentration (pg/ml) median (IQR)	36.4 (7.4)	34.3 (11.7)	0.138
Comparison across genders (p value)	0.954	0.286	
< 40 years CXCL12 concentration (pg/ml) median (IQR)	37.5 (5.4)	35.9 (10.4)	0.183
≥ 40 years CXCL12 concentration (pg/ml) median (IQR)	35.5 (11.1)	34.1 (9.2)	0.572
Comparison across age groups (p value)	0.114	0.557	

IQR – interquartile range, MS – multiple sclerosis.
